# Novel type of references for BMI aligned for onset of puberty – using the QEPS growth model

**DOI:** 10.1186/s12887-022-03304-3

**Published:** 2022-04-30

**Authors:** Kerstin Albertsson-Wikland, Aimon Niklasson, Lars Gelander, Anton Holmgren, Andreas F. M. Nierop

**Affiliations:** 1 Department of Physiology/Endocrinology, Institute of Neuroscience and Physiology, Sahlgrenska Academy, University of Gothenburg, SE 405 30 Gothenburg, Sweden; 2 Göteborg Pediatric Growth Research Center, Department of Pediatrics, Institute of Clinical Sciences, Sahlgrenska Academy, University of Gothenburg, Gothenburg, Sweden; 3 Department of Pediatrics, Halmstad Hospital, Halmstad, Sweden; 4 Muvara bv, Multivariate Analysis of Research Data, Leiderdorp, Netherlands

**Keywords:** Pubertal growth, Personalized growth, Biological age, SDS, Standard reference population

## Abstract

**Objectives:**

Despite inter-individual variations in pubertal timing, growth references are conventionally constructed relative to chronological age (C-age). Thus, they are based on reference populations containing a mix of prepubertal and pubertal individuals, making them of limited use for detecting abnormal growth during adolescence. Recently we developed new types of height and weight references, with growth aligned to age at onset of the pubertal growth spurt (P-age). Here, we aim to develop a corresponding reference for pubertal BMI.

**Methods:**

The QEPS-height and weight models were used to define a corresponding QEPS-BMI model. QEPS-BMI was modified by the same individual, constitutional weight–height-factor (WHF) as computed for QEPS-weight. QEPS-BMI functions were computed with QEPS weight and height functions fitted on longitudinal measurements from 1418 individuals (698 girls) from GrowUp_1990_Gothenburg cohort. These individual BMI functions were used to develop BMI references aligned for height at AgeP5; when 5% of specific puberty-related (P-function) height had been attained. Pubertal timing, stature at pubertal onset, and childhood BMI, were investigated in subgroups of children from the cohort GrowUp_1974_Gothenburg using the new references.

**Results:**

References (median, standard deviation score (SDS)) were generated for total BMI (QEPS-functions), for ongoing prepubertal growth (QE-function) vs C-age, and for total BMI and separated into BMI specific to puberty (P-function) and BMI gain from ongoing basic growth (QES-functions), allowing individual growth to be aligned based on P-age. Growth in basic BMI was greater than average for children categorized as tall and/or with high-BMI at puberty-start. In children categorized as short at puberty-start, P-function-related-BMI was greater than average.

**Conclusions:**

Use of these new pubertal BMI references will make it possible for the first time to consider individual variations owing to pubertal timing when evaluating BMI. This will improve the detection of abnormal changes in body composition when used in combination with pubertal height and weight references also abnormal growth. Other benefits in the clinic will include improved growth monitoring during treatment for children who are overweight/obese or underweight. Furthermore, in research settings these new references represent a novel tool for exploring human growth.

**Supplementary Information:**

The online version contains supplementary material available at 10.1186/s12887-022-03304-3.

## Background

For many decades, body mass index (BMI) has been the most commonly used variable to define and evaluate body composition and weight status in children. In both research and clinical practice, BMI, which is expressed as kg/m^2^, has been widely used to define whether a child is of normal weight, underweight, overweight, or have obesity [1]. While both height and weight increase with chronological age (C-age), this is not the case for BMI. BMI rises in infancy, then falls during childhood before rising again [2–4]. The second rise in BMI is usually seen some years before puberty and continues during the adolescent years, with BMI only reaching adult levels sometime after adult height has been attained. In 2012, the international obesity task force (IOTF) used merged BMI data from different countries to create a worldwide-accepted reference with iso-lines defining overweight, obesity, and thinness [5], which has been especially valuable for international comparison of research studies. Beside this, there are national BMI references, such as those in Sweden which include risk estimation curves for obesity [3].

The ongoing obesity epidemic complicates the construction of new weight and BMI references. Although the trend over time toward higher BMI seems partly to have plateaued in some high-income countries, BMI is still rising in many parts of the world [6]. Recently in Sweden, for example, an increasing prevalence of overweight and obesity with age in both girls and boys has been reported [7]. Such changes in BMI must not be incorporated in references reflecting optimal body composition. We addressed this issue in the recently updated Swedish references for weight and BMI by omitting individuals with obesity from the longitudinal BMI dataset used to develop the references [4].

It has been convention for growth references for height, weight, and BMI to be presented in relation to C-age, making them suboptimal for detecting abnormal growth during the period of adolescence which is characterized by great inter-individual variations in growth because of differences between individuals in the C-age at which they enter puberty [8, 9]. When in 2020 developing the most recent Swedish height references, to account for this we included a ‘prepubertal’ reference showing the growth that during adolescence is continuing independently of puberty [10]. Unlike the previous reference developed around 2000 in Sweden that have used estimates of ongoing, childhood growth from the Infancy–Childhood–Puberty (ICP) growth model [11–13], we constructed prepubertal growth using the QE-functions of the QEPS-model [14, 15]. Individual growth is described by the QEPS-model using four mathematical functions: a Q (Quadratic) and E (Exponential) function arising before birth and resulting in the prepubertal growth, to which a specific pubertal growth function, P, is added; the QE-growth is during puberty transformed and ended by a S (stop) function; resulting in basic, QES-function-growth during puberty [14]. Thereafter, we also developed a QEPS-model for weight and constructed a reference for prepubertal weight gain during adolescence [16].

Recently, we have developed a new type of pubertal height reference describing growth aligned for onset of the specific P-function in the QEPS-model responsible for pubertal height gain; this reference provides information on total growth, as well as separating out the specific growth related to puberty from basic growth continuing during the pubertal years [10, 17]. Subsequently, we developed a QEPS model for weight to construct corresponding references for pubertal weight gain [16]. It now remains to develop a puberty-adjusted growth model for BMI and to use it to develop references that enable the separation of prepubertal and pubertal changes in BMI.

The aim of this study was to use the QEPS-models for height and weight to develop a growth model for BMI, and to develop new BMI references that take biological maturation of the individual during adolescence into account. Like the previous references, a highly selected reference population was used representing healthy children’s growth, i.e., how children ought to growth. Data used to construct the new BMI reference was obtained from longitudinally followed, healthy children born at term to non-smoking mothers and Nordic parents, selected from the GrowUp_1990_Gothenburg cohort born in Sweden [4]. Separate references will be constructed for (a) prepubertal BMI (Q- and E-functions), (b) total BMI (modeled by the Q, E, P and S-functions), (c) BMI gain specific to puberty (by the P-function), and (d) BMI gain that is not specific to puberty (basic BMI, by the Q, E, and S-functions). Subgroups of children from the GrowUp_1974_Gothenburg cohort categorized based on the timing of puberty (early, average, late), height at start of puberty (tall, short) or body composition during childhood (high BMI, low BMI) will be used to explore the utility of the new references for monitoring BMI.

## Materials and methods

### Ethical approval

Ethical approval was obtained from the Regional Ethics Review Board in Gothenburg (Ad 91–92/131–93 and Ad 444–08 T062–09). Informed consent was given by participants and parents of individuals < 18 years of age. Studies were conducted in accordance with the principles of the Declaration of Helsinki.

### Materials

#### Reference population from GrowUp_1990_Gothenburg cohort

In total, 1418 individuals (698 girls) from the GrowUp_1990_Gothenburg cohort were included in the cohort used to construct the QEPS-BMI reference. This was the same population as used to develop earlier C-age references for total weight and BMI, and those for weight-for-height (see Supplemental Table S1 in Albertsson-Wikland et al. [4]). The cohort included only healthy children (see Table 1 in Albertsson-Wikland et al. [4]) who had Nordic parents and were born in Sweden around 1990 at full term (gestational age (GA) 37–43 weeks) to non-smoking mothers. Information was available on longitudinal growth until adult height for all participants. For more information see Albertsson-Wikland et al. [4, 17].

#### Subgroups in the GrowUp_1974_Gothenburg cohort used for exploring the novel reference

Evaluation of the utility to research of the new pubertal BMI reference was made using data from healthy children from the GrowUp_1974_Gothenburg cohort (2177 subjects: 1081 girls). The impact of grouping children according to pubertal age defined as age at peak height velocity (PHV) for the total growth curve, AgeTPHV, as early, <− 1.5 yrs., average, ±0.25 yrs., and late, > + 1.5 yrs.; for height at onset of puberty as tall > + 1.5SDS, short < − 1.5SDS, and for childhood BMI as high > 1.5SDS, low <− 1.5SDS, was explored (see Supplemental Table S3 of Albertsson-Wikland et al. [10]).

### QEPS-BMI method

The QEPS-height [14] and QEPS-weight models [16], were used to define a corresponding QEPS-BMI model. The QEPS-BMI model is expressed in kg^0.5^/m to be consistent with the QEPS-weight model, which was expressed in kg^0.5^ to enable the additive property of the QEPS weight functions. Where appropriate, we show BMI in kg/m^2^ in figures on the left axis and BMI in kg^0.5^/m on the right axis. For detailed information about the QEPS-BMI model see Supplement, including Supplemental Figures S1, S2, and S3. Like the QEPS-weight model, QEPS-BMI was modified by an individual ‘constitutional factor’, a weight–height factor (*WHF*): *WHF* = 0, a ‘normal body constitution’, *WHF* > 0, a heavy, and *WHF* < 0, a lean body constitution. Traditional references according to C-age for total and prepubertal BMI were computed in two steps comparable to the ‘QEPS method used for the references’ section in Supplement of Albertsson-Wikland et al. [17], but with QEPS height functions replaced by corresponding QEPS-BMI functions. QEPS-BMI functions were computed with QEPS-height functions fitted on height measurements and QEPS-weight functions fitted on weight measurements while including information from fitted QEPS-height functions.

Comparison was made between the total BMI reference obtained using the QEPS-BMI method and the previously published BMI reference obtained by applying the LMS method [4]. Both references had been created using the same data from the same population, see Supplemental Figure S4.

Median curves for the QEPS-derived and LMS-derived references were similar; however, as expected, variance was smaller for the QEPS-derived reference owing to computation with fitted functions that excluded residual variation. A new reference for pubertal BMI was then generated. The onset of puberty was identified based on height-specific *P*-function growth; the point at which 5% of height *Pmax* had been obtained (*AgeP5*) was used to define the onset of puberty (Supplemental Figure S2 in Albertsson-Wikland et al.) [10, 15]. Height for each individual was then aligned according to age at the onset of puberty. To achieve this for BMI, all individual longitudinal QEPS-BMI-functions were aligned according to height at *AgeP5.* For more information see Supplement.

### Statistical evaluations

Longitudinal growth data for each individual were exported to Matlab® (version 9.3 R2017b, The MathWorks). Estimation of individual QEPS height and weight parameters by nonlinear fitting was conducted using the Matlab Curve Fitting Toolbox. The fitting procedure for height is described in [14], and for weight in the Supplement [16]. QEPS-BMI functions were computed with resulting QEPS height and weight functions. To simplify general mathematical formulae, age was assumed to be equal to age corrected for GA, here 37–43 weeks. All figures were prepared in Matlab; Fig. 7 was finalized in Photoshop.

## Results

### Total and prepubertal BMI references vs chronological age (C-age)

**Fig. 1:**
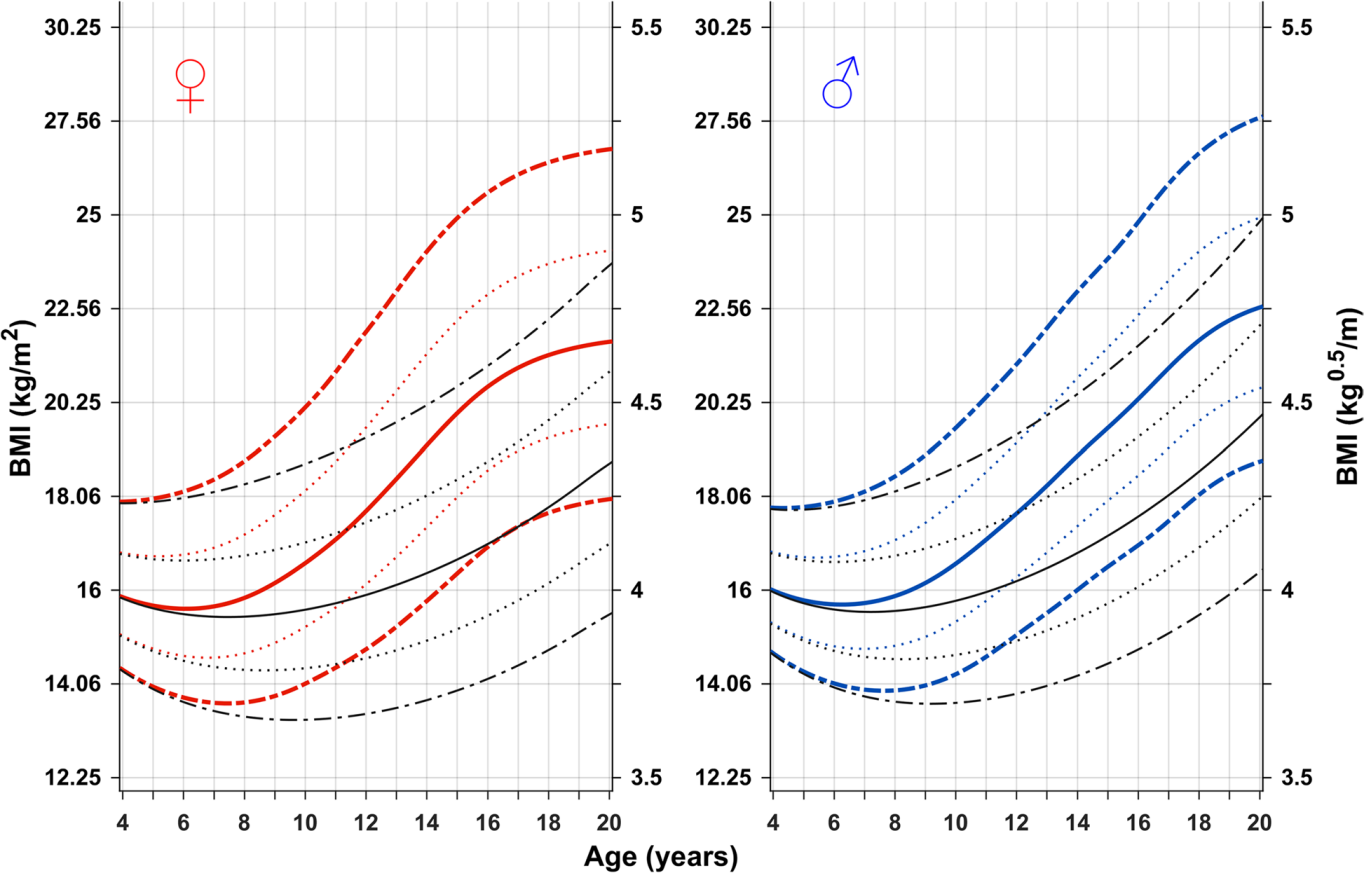
Chronological age reference for total BMI and prepubertal BMI (QE-function)) for girls (left) and boys (right) aged 4–20 years. Median *total BMI* (red/blue solid line, ±1SDS (red/blue dotted line)), and ± 2SDS (red/blue dashed line) and median *prepubertal BMI* (black)

The novel BMI references in kg/m^2^ (left axis) and in kg^0.5^/m (right axis) according to C-age 4–20 years for girls and boys, Fig. 1. Total BMI is shown in color (red/blue), and BMI gained independently of puberty, the prepubertal, (QE-function) BMI reference, in black.

### BMI references aligned for onset of puberty (P-age)

**Fig. 2:**
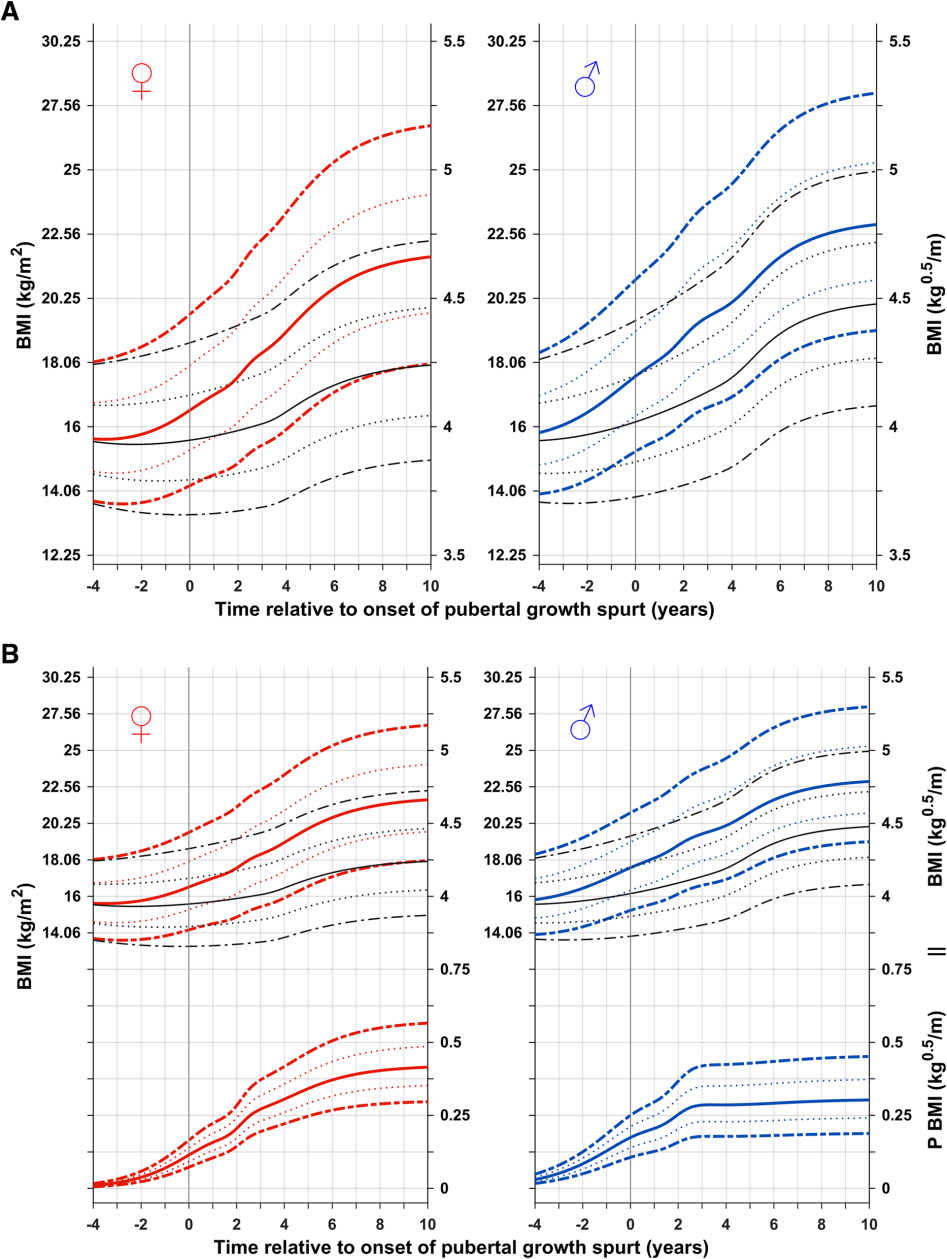
Upper panel: References in kg/m^2^ (left axis) and in kg^0.5^/m (right axis) for total BMI and the basic BMI (BMI gained independently of puberty (QES-function) for girls (red, left) and boys (blue, right). Curves are aligned for age at onset of pubertal height spurt (AgeP5). Median *total BMI* (red/blue solid line, ±1SDS (red/blue dotted line), and ± 2SDS (red/blue dashed line)) and median *basic BMI* (black). General differences in timing between pubertal height and weight functions result in an undulation of aligned BMI functions. Lower panel: BMI gain resulting from the P-function of the QEPS (P-BMI) provides a puberty-specific BMI reference. *Specific P-function-derived BMI* as median, ±1SDS (dotted line), and ± 2SDS (dashed line). The individual onset of puberty was identified and aligned based on the age at which 5% (AgeP5) of the total specific P-function growth (Pmax) for height had occurred. General differences in timing between pubertal height and weight functions result in an undulation of aligned BMI functions

The reference shown in Fig. 2 is for total (QEPS) and basic (QES-function) BMI in kg/m^2^ (left axis) and kg^0.5^/m (right axis) for girls and boys aligned according to the onset of puberty, estimated as AgeP5 for height, P-age. Specific P-function growth has been included in the lower panel. BMI references in both figures are depicted from 4 years before to 10 years after the onset of the pubertal growth spurt to capture changes in BMI relative to the acceleration in height and weight that occurs during puberty. General differences in the timing of pubertal height and weight functions resulted in undulations of the aligned BMI functions (Supplemental Figure S3).

### Rational for a puberty-aligned BMI reference

**Fig. 3:**
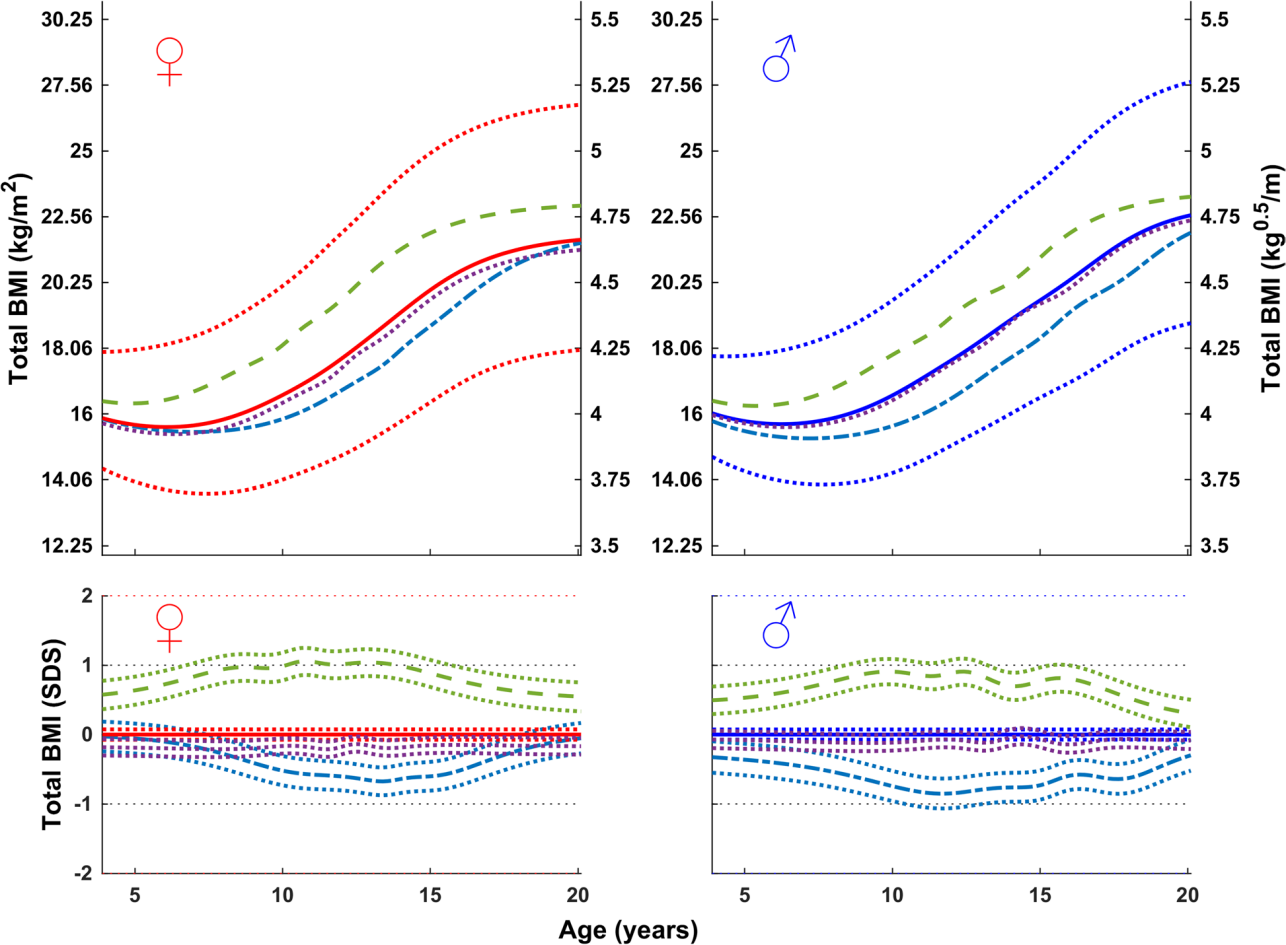
Median total BMI (in kg/m^2^, top; in SDS with 95% CI, bottom) according to chronological age for girls (left) and boys (right) from the GrowUp_1974_ Gothenburg cohort grouped according to timing of puberty: early, <− 1.5 yrs. (− − −), average, ±0.25 yrs. (• • •), and late, > + 1.5 yrs. (− − −). Data are visualized alongside the reference for median total BMI (thick solid lines) and ± 2SDS (dotted red (left) and blue (right) lines)

Total BMI relative to C-age for girls and boys with an early, average, and late onset of puberty is shown in Fig. 3. As expected, BMI gain occurred sooner than average in children with an early and later than average in children with a late onset of puberty. This highlights the inadequacy for many adolescents of using a C-age-based BMI reference.

### Exploring pubertal BMI in subgroups from the GrowUp_1974_Gothenburg cohort

#### Timing of puberty

**Fig. 4:**
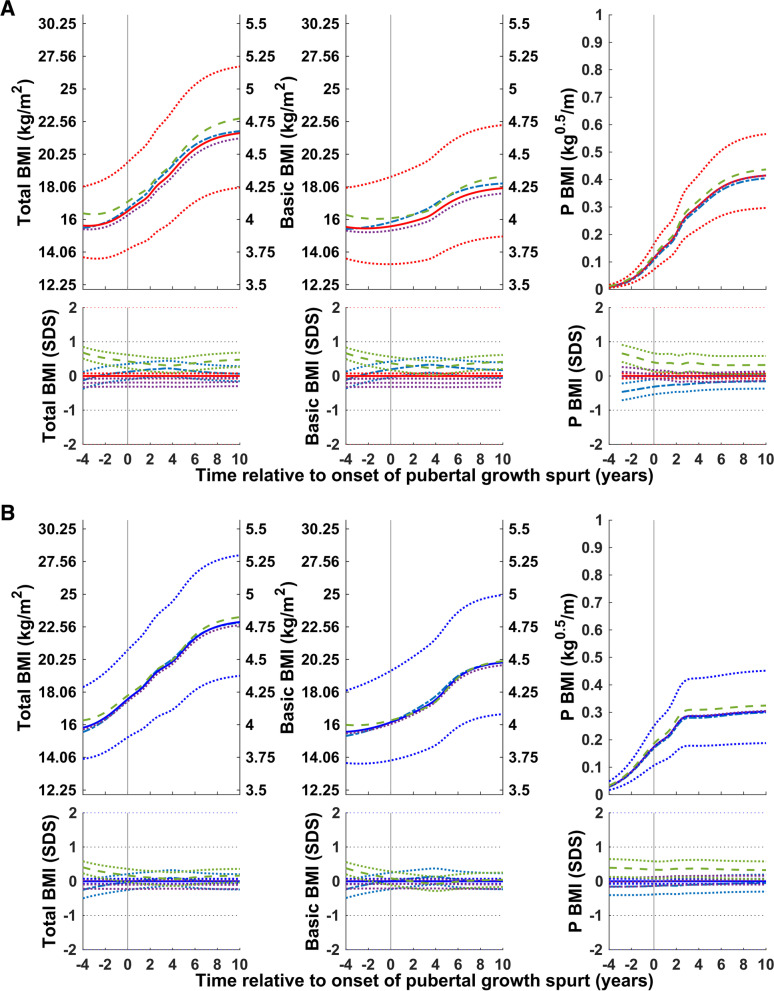
Median total, basic, and puberty-specific BMI gain (in kg/m^2^, top; in SDS with 95% CI, bottom) relative to onset of puberty for girls (red at the top) and boys (blue at the bottom) grouped according to timing of puberty: early, <− 1.5 yrs. (− − −), average, ±0.25 yrs. (• • •), and late, > + 1.5 yrs. (− − −). Median total BMI (QEPS-functions, left panels), basic BMI growth (QES-functions, middle panels), and specific pubertal BMI growth (P-function, right panels) from the GrowUp_1974_Gothenburg cohort. Curves are aligned for age at onset of pubertal growth spurt. Data are visualized alongside the new reference for median total BMI (thick red or blue solid lines) and ± 2SDS (red/blue dotted lines)

Total (QEPS), basic (QES), and pubertal (P-function) BMI relative to the onset of puberty for subgroups of girls and boys with an early, average, and late onset of puberty is shown in Fig. 4. For girls in the early and late puberty onset groups, the total BMI gain and the gain in the basic component of BMI were somewhat higher than average; for boys in the early and late puberty onset groups, gains in total and basic BMI were similar to average. Puberty-specific BMI gain was greater than average in the early-puberty-onset group and lower than average in the late-puberty-onset group.

#### Stature

**Fig. 5:**
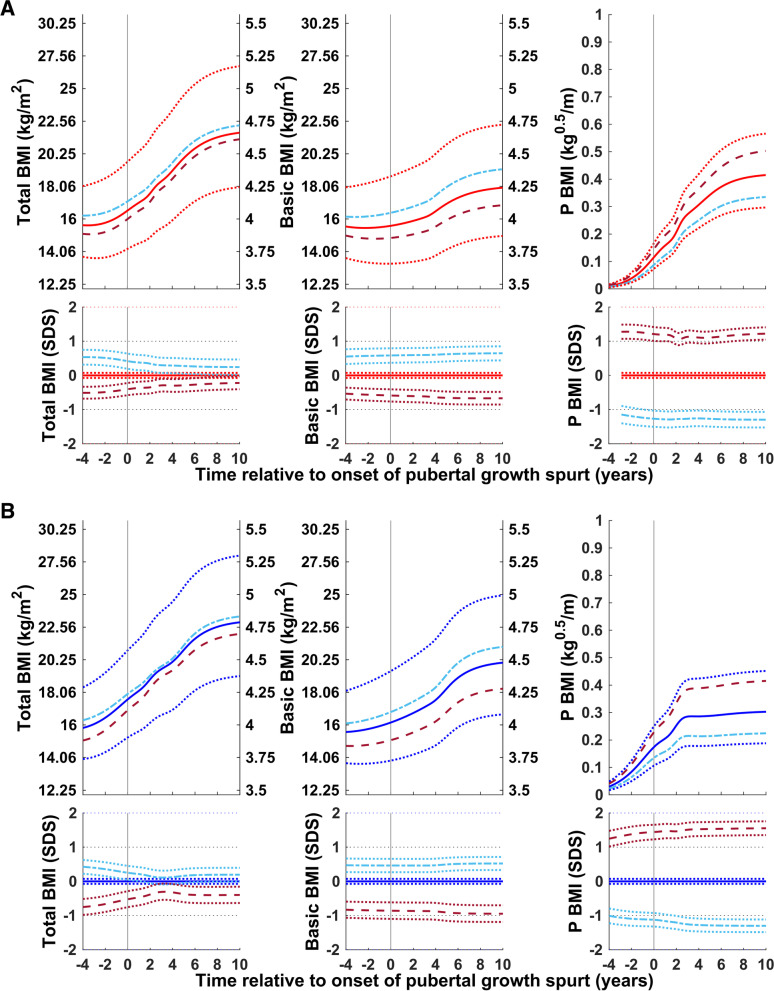
Median total, basic, and puberty-specific BMI gain (in kg/m^2^, top; in SDS with 95% CI, bottom) relative to the onset of puberty for girls (red at the top) and boys (blue at the bottom) according to height at the onset of puberty: tall, > + 1.5 SDS (− − −) and short, <− 1.5 SDS (− − −). Median total BMI (QEPS-functions, left panels), basic BMI growth (QES-functions, middle panels), and specific pubertal BMI growth (P-function, right panels) from the GrowUp_1974_Gothenburg cohort. Curves are aligned for age at onset of pubertal growth spurt. Data are visualized alongside the new reference for median total BMI (thick red or blue solid lines) and ± 2SDS (red/blue dotted lines)

The total (QEPS-functions), basic (QES-functions), and pubertal (P-function) BMI for girls and boys according to height at P-age, the age at the onset of puberty is shown in Fig. 5. For girls and boys who were tall at the onset of puberty, the basic component of BMI was higher than average; for girls and boys who were short at the onset of puberty, this component was lower than average. In contrast, puberty-specific BMI gain was lower than average for the tall group and higher than average for the short group.

#### Degree of childhood BMI

**Fig. 6:**
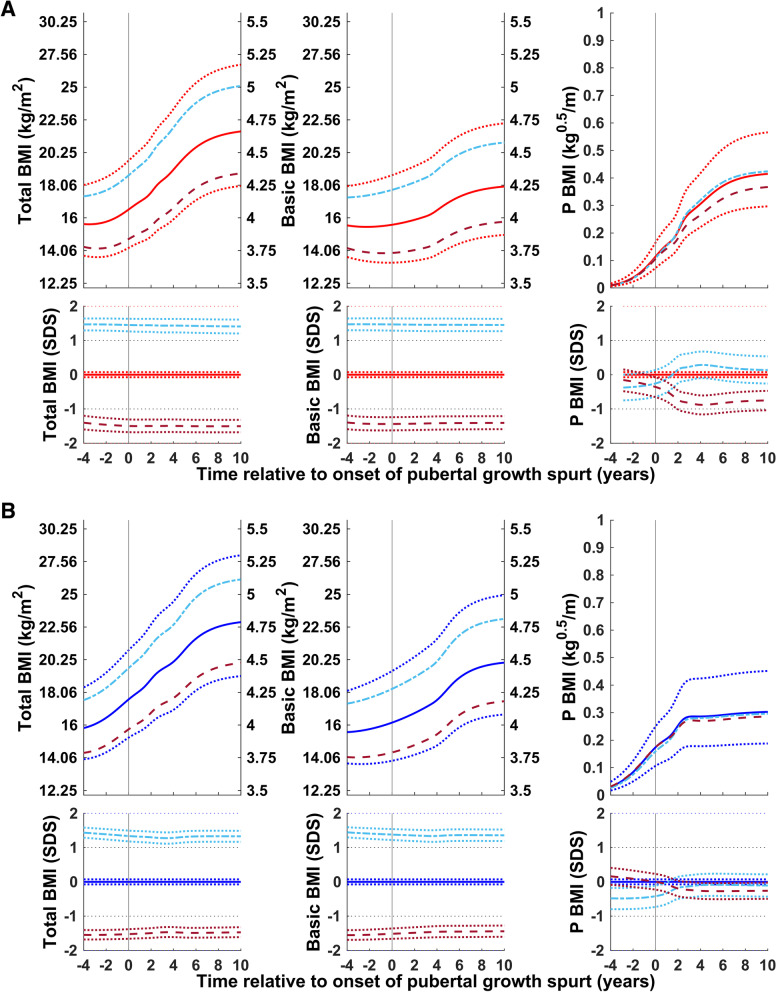
Total, basic, and puberty-specific BMI gain (in kg/m^2^, top; in SDS with 95%CI, bottom) relative to the onset of puberty for girls (red at the top) and boys (blue at the bottom) according to BMI in childhood: high BMI, > + 1.5SDS (− − −) and low BMI, <− 1.5SDS (− − −). Median total BMI (QEPS-functions, left panels), basic growth (QES-functions, middle panels), and specific pubertal growth (P-function, right panels) in girls and boys from the GrowUp_1974_Gothenburg cohort. Curves are aligned for age at onset of pubertal growth spurt. Data are visualized alongside the new reference for median total BMI (thick red or blue solid lines) and ± 2SDS (red/blue dotted lines)

Total (QEPS-functions), basic (QES-functions), and pubertal (P-function) BMI for girls and boys according to the highest BMI during childhood (girls:3.5–7 yrs.; boys: 3.5–8 yrs), Fig. 6. Gains in the basic component of BMI were higher than average in children in the high BMI group at the onset of puberty and lower than average in the low BMI group. In the low BMI groups, puberty-specific BMI gain was lower than average for girls and somewhat lower than average for boys. In the high BMI groups, puberty-specific BMI gain was somewhat higher than average for girls and similar to the average for boys.

### Using the new BMI pubertal growth charts prospectively for an individual child

**Fig. 7:**
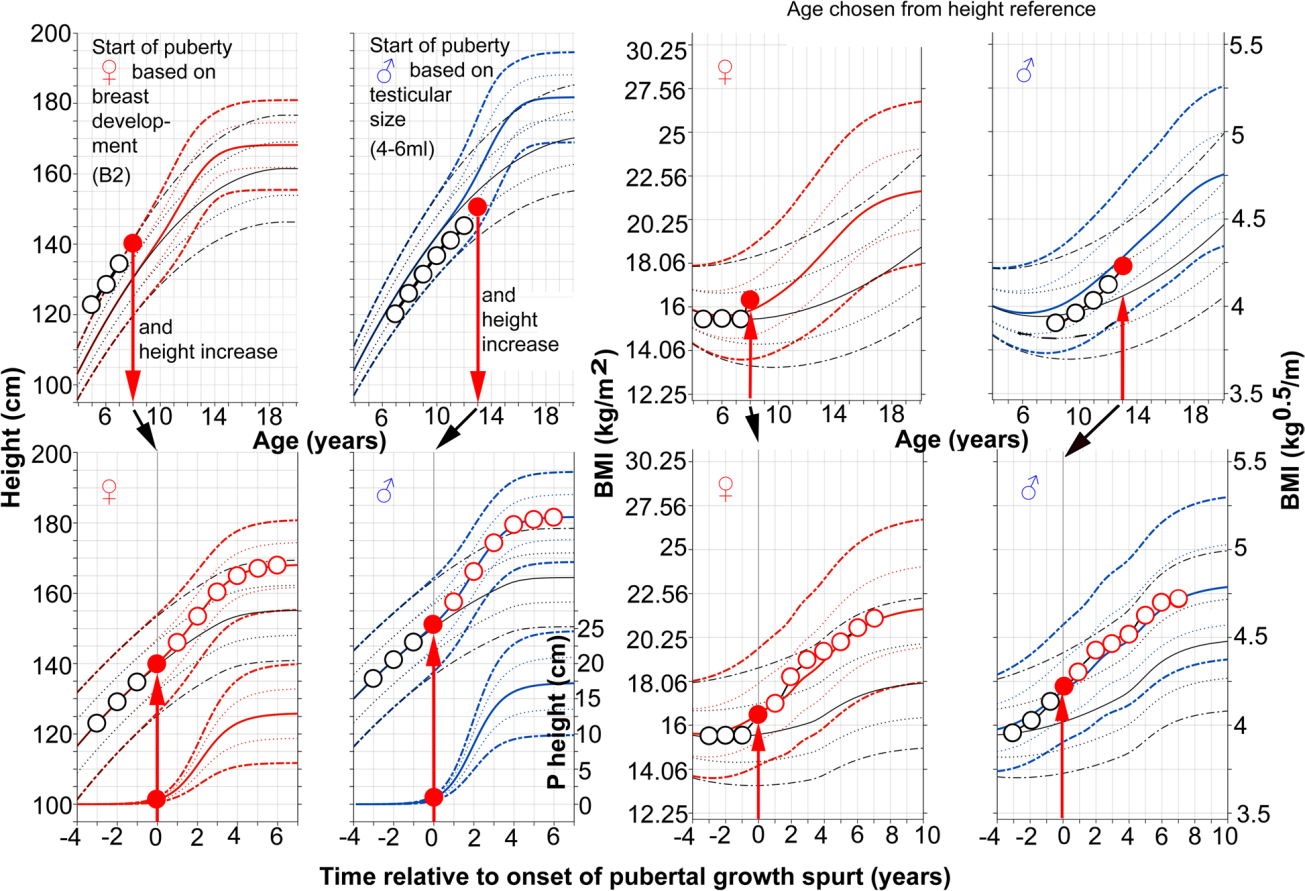
Guide for prospective use of the pubertal-age-adjusted reference for total BMI (kg/m^2^ and kg^0.5^/m) and total height (cm) for girls (♀) and boys (♂). Individual age adjustment is made using only the height measurement at the chronological age (C-age) at which puberty started (see upper left traditional C-age-reference for girls and boys) [14], by using the height increase from the individual prepubertal growth curve (as drawn in upper left panel) through the individual measuring points. This measurement is usually corresponding to the time when secondary sex characteristics develop; early breast development stage, B2 in girls (red, upper left panel), or testicular volume increase to 4–6 ml in boys (blue, upper left panel). Individual growth curves are shown for a girl aged 8 years and a boy aged 13-years at the start of puberty [14]. Height and BMI according to C-age and P-age, i.e. the age adjusted to reflect the start of puberty, are shown for an 8-year-old girl and a 13-year-old boy [14]. Height (cm) and BMI (in kg/m^2^ or kg^0.5^/m) according to C-age at the onset of puberty, and to P-age, after adjustment for age at onset of the pubertal growth spurt, are depicted as red dots (all panels), based only on age in the height references for each sex, respectively. Corresponding heights and BMIs are then moved to puberty-adjusted age = zero, in the P-age-references shown in the lower panels (left panels for height and right for BMI). Thereafter, all measured heights and BMIs are depicted at ages/times recalculated in relation to the specific onset of puberty in that individual, labelled on the x-axis as ‘Time from onset of growth spurt (years)’. Thus, changes in BMI and height in the years preceding the pubertal growth spurt can be evaluated using the novel references. Note, the BMI increase precedes the height increase in relation to puberty. For corresponding values for weight, see Fig. 7 in Albertsson-Wikland et al. [16]

In order to monitor pubertal BMI changes prospectively for an individual child using the new pubertal-age-BMI charts a manual procedure can be undertaken. Early identification of the ‘take-off’ in growth at the start of puberty can be achieved using the growth charts for total and prepubertal height references [17]. Figure 7 describes the prospective use of the pubertal-age-adjusted reference to assess total BMI. For corresponding changes in weight, see Fig. 7 in Albertsson-Wikland et al. [16].

## Discussion

### New prepubertal and pubertal BMI references

Here, we present the first pubertal BMI references adjusted for biological maturation based on age at onset of the pubertal growth spurt in the individual child. Thereby, the growth of any individual will be related only to peers with similar biological age and maturation. Another novel benefit of these BMI references is that they allow the separation of prepubertal and pubertal components of BMI gain. During adolescence this makes it possible to look in detail at the ongoing BMI gain that is unrelated to puberty in relation to the growth specific to puberty. As such, we expect these references to be of utility in the clinic and in research-based investigations of both puberty-independent and puberty-dependent changes in BMI. Moreover, a prepubertal BMI reference was also created, vs C-age, which will be particularly important when considering body composition as BMI changes in children in whom puberty is early or delayed.

These new references complete a set of three growth references – for pubertal and prepubertal height, weight, and BMI – developed using growth functions based on QEPS growth models for height, weight, and BMI. In all cases, these references make it possible to align growth in the individual relative to the onset of pubertal growth in the individual [10, 16] and as a set, they will be valuable in pubertal and prepubertal individuals for evaluating growth in terms of height, weight, and BMI, as well as for evaluating the relationship between weight and height. To use growth references such as these effectively in healthcare and clinical settings, estimation of the stage of pubertal maturation will be required alongside construction of the growth chart for the individual; this includes investigation of the genitalia and breast maturation in girls or testicular size in boys [18]. Using these three references alongside estimates of pubertal maturation will help to decide which reference for each variable to use, and will improve the precision with which we can evaluate changes in BMI and growth during adolescence [10].

### BMI changes preceding height changes

The occurrence of characteristic sex-specific changes in weight and body fat mass before changes in pubertal growth suggest a relationship between energy storage in adipose tissue and pubertal maturation [19]. By aligning BMI in relation to the onset of puberty, the new BMI references will allow us to explore in detail the changes in body composition (BMI and weight) that occur some years before the increase in height associated with puberty (Supplemental Figure S3).

It is interesting to note that the BMI reference curves were observed to be undulating in places. This appears to be due to differences in the timing of puberty-related changes in height and weight, and to the type of tissue growth responsible for weight gain (e.g. fat vs muscle). Thus, residual variation around individual predicted BMI for normal body constitution might be related, not only to measuring errors, but also to individual variations in the relationship of fat and muscle distributions as estimated by body compartment analyses (dual-energy X-ray absorptiometry (DEXA)) or other comparable measurements [20]. Previously, we found sex differences in both the timing and amount of two types of weight: earlier gain of type A weight in girls and greater gain of type B weight in boys (Supplemental Figure S3). At present, we speculate that type A weight represents fat tissue because it correlates with the sex difference in the timing of early pubertal fat gain, as described in the study by Vizmanos & Marti-Henneberg, and that type B weight represents muscle tissue; however, this needs to be confirmed in future studies [19, 21]. Puberty-aligned BMI represents a relative balance between weight and height, making interpretation of these BMI results quite complex. Interestingly, the total BMI typical mean function for individual boys and girls became quite similar over time, whereas the P-function-related curve showed sex differences in both the timing and proportion of pubertal type A BMI gain and pubertal type B BMI gain (Supplemental Figure S3). DEXA studies will be needed to explore this observation in greater detail.

### Usefulness of a reference separating BMI growth functions during puberty

The benefits of using a growth model to explore underlying growth regulating mechanisms have been discussed previously [16, 22, 23]. A novel finding from our analysis of subgroups of children with different characteristics prior to entering puberty was that gain in basic BMI was greater in tall boys and girls, when compared to either the reference, or to boys and girls of average pubertal timing or stature. In contrast, lower-than-average basic BMI gain was observed for children who were short at the start of puberty, although their P-function-related BMI gain was greater than average. This is consistent with our previous investigations in children with different BMIs in which we found a positive relationship between BMI and greater than average gains in basic height and lower than average gains in puberty-specific height [24]. We also found that a high BMI was associated with greater than average gains in basic BMI and that low BMI was associated with lower-than-average increases in basic BMI.

From a research perspective, the new BMI reference will be useful in the exploration of correlates for predicting future health. It is already well known that childhood obesity is an important factor associated with increased risk for reduced life expectancy. For example, increased BMI during adolescence is associated with elevated cardiovascular morbidity, the development of type 2 diabetes and different types of cancer during adult life [25–27]. It is also known that both prenatal and early postnatal nutrition have epigenetic effects on developmental programming resulting in cardiovascular diseases, diabetes, overweight, and obesity [28–30]. As such, the ability to look at the different components of BMI during childhood will be useful for exploring which variables help us to predict the development of obesity.

### National growth screening and improved BMI monitoring during treatment

Longitudinal follow up of children’s growth provides an important way of evaluating present and future population health and should be conducted at a national level [31]. National monitoring of BMI-related child health will be more precise with the incorporation of insights from the BMI references described here. We anticipate that investigations using the new references will lead to re-evaluation of currently used BMI iso-line cutoffs especially during the pubertal years.

With the exception of surgical treatment during late adolescence [32, 33], interventions to treat obesity during childhood are still generally unsuccessful [32]. There is, therefore, a clear need for more clarity on the underlying causes of childhood obesity so that appropriate treatments can be developed. Gaining a greater understanding of the relationship of BMI to puberty will be an important step in this journey. Another condition where more in-depth understanding of BMI will be useful, is anorexia nervosa. Anorexia nervosa often occurs during the adolescent period, and monitoring severity of the disease and its treatment will benefit from using the new puberty onset aligned references for height, weight, and BMI [34–36]. The tool that we present here for investigating BMI changes in new ways and with higher precision than previously possible will be of great utility in these situations.

### A selected reference population for making near standards

A highly selected reference population was used, aiming to represent how healthy children ought to grow, and to reflect healthy BMI, and thereby facilitating global usefulness of the references [4, 10, 16, 17]. Therefore, only healthy children born at term, avoiding the different postnatal growth of premature infants, to nonsmoking mothers, avoiding influence of nicotine on growth, and used as a proxy for low socioeconomic. Only children to Nordic parents, since the positive secular trend in height nearly ended in this region of the world, to minimize the influence of different pace of secular trends related to differences in geographical origin [17]. Population height has been proposed to change in relation to environmental factors but unlikely to change in relation to genetics, if not due to migration [37]. It can be seen as a limitation, only using individuals with Nordic ethnicity as the reference population; especially acknowledging the adult ethnicity-specific BMI cut offs for obesity based on type 2 diabetes risk for different populations [38]. However, on the other hand, this makes it necessary in order to evaluate the relation to disease in a corresponding population, to restrict the reference population to one ethnic group, and not make it arbitrary. As described earlier, we omitted individuals with severe obesity, in order not to follow the increase in weight and BMI with the obesity epidemic [4, 16]. However, growth of any child must be adjusted for mid parental height, and thereby take into consideration both the genetical influence and the pace of secular trend for the individual child.

### Limitations with using BMI

One of the limitations associated with using BMI as a measure of body composition in growing individuals is that the correlation between BMI (w/h^2^) and height has been shown to change with C-age, especially during adolescence. Similarly, other relationships between weight and height vary with age, see Fig. 2 in Karlberg & Albertsson-Wikland, where a beta-factor was introduced [2]. When developing the current QEPS-BMI model we therefore implicitly applied the nonlinear regression coefficients within the QEPS weight model to allow a similar correlation to be maintained across different age ranges. This indicates that the standard formula for calculating BMI is not optimal for describing weight status during childhood and puberty [2].

Human weight represents the sum of fat, muscle, body water, visceral organs, and bones in an individual. Despite the common use of BMI as a measure of adiposity or thinness, BMI cannot differentiate between these different tissues. A further limitation of using BMI as a measure of adiposity is that, while BMI correlates with fat mass in children on a group level, there is not always a correlation at the individual level [39, 40].

## Conclusion

We present new BMI references that can be used to evaluate prepubertal BMI in relation to chronological age, as well as assessing pubertal and prepubertal changes in BMI relative to the onset of the pubertal growth spurt in the individual. When used together with comparable references developed using the QEPS-models for height and weight [10, 17], these novel BMI references will facilitate improved detection and monitoring of growth abnormalities during adolescence within the health care system. Furthermore, in research settings these new references represent a novel tool for exploring human growth. The availability of this type of reference is an important step towards more meaningful and informative clinical monitoring of BMI development of the individual during the adolescent years.

## Supplementary Information

**Additional file 1.**

## Data Availability

The data generated and analyzed during the current study are not publicly available due to that these data are part of several ongoing studies and will be publicly available at a later point in time but are available from the corresponding author on reasonable request.

## References

[1] Holmgren A (2022). The quadratic-exponential-pubertal-stop model is valid for analysing human growth patterns and developing novel growth references. Acta Paediatr.

[2] Karlberg J, Albertsson-Wikland K (1996). Nutrition and linear growth in childhood. Recent developments in infant nutrition.

[3] Karlberg J, Luo ZC, Albertsson-Wikland K (2001). Body mass index reference values (mean and SD) for Swedish children. Acta Paediatr.

[4] Albertsson-Wikland K, Niklasson A, Gelander L, Holmgren A, Sjoberg A, Aronson AS (2021). Swedish references for weight, weight-for-height and body mass index: the GrowUp 1990 Gothenburg study. Acta Paediatr.

[5] Cole TJ, Lobstein T (2012). Extended international (IOTF) body mass index cut-offs for thinness, overweight and obesity. Pediatr Obes..

[6] Collaboration NCDRF (2017). Worldwide trends in body-mass index, underweight, overweight, and obesity from 1975 to 2016: a pooled analysis of 2416 population-based measurement studies in 128.9 million children, adolescents, and adults. Lancet..

[7] Bygdell M, Celind J, Lilja L, Martikainen J, Simonson L, Sjogren L (2021). Prevalence of overweight and obesity from 5 to 19 years of age in Gothenburg, Sweden. Acta Paediatr.

[8] Cole TJ (2012). The development of growth references and growth charts. Ann Hum Biol.

[9] Tanner JM, Whitehouse RH, Takaishi M (1966). Standards from birth to maturity for height, weight, height velocity, and weight velocity: British children, 1965. I. Arch Dis Child.

[10] Albertsson-Wikland KG, Niklasson A, Holmgren A, Gelander L, Nierop AFM (2020). A new type of pubertal height reference based on growth aligned for onset of pubertal growth. J Pediatr Endocrinol Metab.

[11] Karlberg J (1987). On the modelling of human growth. Stat Med.

[12] Karlberg J, Fryer JG, Engstrom I, Karlberg P (1987). Analysis of linear growth using a mathematical model. II. From 3 to 21 years of age. Acta Paediatr Scand.

[13] Albertsson-Wikland K, Luo ZC, Niklasson A, Karlberg J (2002). Swedish population-based longitudinal reference values from birth to 18 years of age for height, weight and head circumference. Acta Paediatr.

[14] Nierop AF, Niklasson A, Holmgren A, Gelander L, Rosberg S, Albertsson-Wikland K (2016). Modelling individual longitudinal human growth from fetal to adult life - QEPS I. J Theor Biol.

[15] Holmgren A, Niklasson A, Gelander L, Aronson AS, Nierop AFM, Albertsson-Wikland K (2017). Insight into human pubertal growth by applying the QEPS growth model. BMC Pediatr.

[16] Albertsson-Wikland K, Niklasson A, Gelander L, Holmgren A, Nierop AFM (2021). Novel type of references for weight aligned for onset of puberty - using the QEPS growth model. BMC Pediatr.

[17] Albertsson-Wikland K, Niklasson A, Holmgren A, Gelander L, Nierop AFM (2020). A new Swedish reference for total and prepubertal height. Acta Paediatr.

[18] Rollof L, Elfving M (2012). Evaluation of self-assessment of pubertal maturation in boys and girls using drawings and orchidometer. J Pediatr Endocrinol Metab.

[19] Vizmanos B, Marti-Henneberg C (2000). Puberty begins with a characteristic subcutaneous body fat mass in each sex. Eur J Clin Nutr.

[20] Karlsson AK, Kullberg J, Stokland E, Allvin K, Gronowitz E, Svensson PA (2013). Measurements of total and regional body composition in preschool children: a comparison of MRI, DXA, and anthropometric data. Obesity (Silver Spring).

[21] Backman G (1940). Gewichtswachstum des Mannes. Wilhelm Roux’ Archiv für Entwicklungsmechanik der Organismen.

[22] Hochberg Z, Albertsson-Wikland K (2008). Evo-devo of infantile and childhood growth. Pediatr Res.

[23] Hochberg Z. Evo-devo of child growth: treatise on child growth and human evolution. 1st ed. New York: Wiley-Blackwell, Publishers; 2012.

[24] Holmgren A, Niklasson A, Nierop AF, Gelander L, Aronson AS, Sjoberg A (2017). Pubertal height gain is inversely related to peak BMI in childhood. Pediatr Res.

[25] Kindblom JM, Bygdell M, Sonden A, Celind J, Rosengren A, Ohlsson C (2018). BMI change during puberty and the risk of heart failure. J Intern Med.

[26] Celind J, Ohlsson C, Bygdell M, Nethander M, Kindblom JM (2019). Childhood body mass index is associated with risk of adult Colon Cancer in men: an association modulated by pubertal change in body mass index. Cancer Epidemiol Biomarkers Prev.

[27] Ohlsson C, Bygdell M, Nethander M, Rosengren A, Kindblom JM (2019). BMI change during puberty is an important determinant of adult type 2 diabetes risk in men. J Clin Endocrinol Metab.

[28] Barker DJ, Osmond C, Kajantie E, Eriksson JG (2009). Growth and chronic disease: findings in the Helsinki birth cohort. Ann Hum Biol.

[29] Eriksson JG, Forsen TJ, Kajantie E, Osmond C, Barker DJ (2007). Childhood growth and hypertension in later life. Hypertension..

[30] Roseboom T, de Rooij S, Painter R (2006). The Dutch famine and its long-term consequences for adult health. Early Hum Dev.

[31] Janson A (2021). The growth of nations’ children. Acta Paediatr.

[32] Hagman E, Danielsson P, Lindberg L, Marcus C, Committee BS (2020). Paediatric obesity treatment during 14 years in Sweden: lessons from the Swedish childhood obesity treatment register-BORIS. Pediatr Obes.

[33] Olbers T, Beamish AJ, Gronowitz E, Flodmark CE, Dahlgren J, Bruze G (2017). Laparoscopic roux-en-Y gastric bypass in adolescents with severe obesity (AMOS): a prospective, 5-year, Swedish nationwide study. Lancet Diabetes Endocrinol.

[34] Modan-Moses D, Yaroslavsky A, Pinhas-Hamiel O, Levy-Shraga Y, Kochavi B, Iron-Segev S (2021). Prospective longitudinal assessment of linear growth and adult height in female adolescents with anorexia nervosa. J Clin Endocrinol Metab.

[35] Leger J, Fjellestad-Paulsen A, Bargiacchi A, Pages J, Chevenne D, Alison M (2021). One year of GH treatment for growth failure in children with anorexia nervosa: a randomized placebo-controlled trial. J Clin Endocrinol Metab.

[36] Berkowitz SA, Witt AA, Gillberg C, Rastam M, Wentz E, Lowe MR (2016). Childhood body mass index in adolescent-onset anorexia nervosa. Int J Eat Disord.

[37] Perkins JM, Subramanian SV, Davey Smith G, Ozaltin E (2016). Adult height, nutrition, and population health. Nutr Rev.

[38] Caleyachetty R, Barber TM, Mohammed NI, Cappuccio FP, Hardy R, Mathur R (2021). Ethnicity-specific BMI cutoffs for obesity based on type 2 diabetes risk in England: a population-based cohort study. Lancet Diabetes Endocrinol.

[39] Mei Z, Grummer-Strawn LM, Pietrobelli A, Goulding A, Goran MI, Dietz WH (2002). Validity of body mass index compared with other body-composition screening indexes for the assessment of body fatness in children and adolescents. Am J Clin Nutr.

[40] Freedman DS, Wang J, Maynard LM, Thornton JC, Mei Z, Pierson RN (2005). Relation of BMI to fat and fat-free mass among children and adolescents. Int J Obes.

