# Supplementary material for: Novel type of references for BMI aligned for onset of puberty – using the QEPS growth model
Source: BMC Pediatr. 2022 Apr 30;22:238. doi: 10.1186/s12887-022-03304-3 (PMC9055717; doi:10.1186/s12887-022-03304-3)
Supplement: Supplementary file 1 — Additional file 1. [file 12887_2022_3304_MOESM1_ESM.pdf]

Supplemental Material

To Albertsson-Wikland K et al: Novel type of references for BMI aligned for onset of puberty – using the QEPS growth model

QEPS BMI (bmiQEPS) growth model page 1

Figure S1: QEPS BMI for normal, heavy and lean body constitution page 2

Figure S2: QEPS height and weight for normal, heavy and lean body constitution page 3

Figure S3: Detailed QEPS pubertal growth estimates for height, weight and BMI page 4

bmiQEPS method for reference development page 4

Figure S4: Total QEPS BMI vs Total LMS BMI page 5

REFERENCES page 5

QEPS BMI (bmiQEPS) growth model

The QEPS height model (1) and weight model (2) were used to define a corresponding QEPS BMI model. The QEPS-model and the corresponding QEPS-functions for height will be called hQEPS-model and hQEPS-functions, for weight they will be called wQEPS-model and wQEPS-functions, and for BMI bmiQEPS-model and bmiQEPS-functions. The bmiQEPS-model for individual growth in BMI was defined by a combination of four growth functions derived from height and weight basic growth functions: a prepubertal quadratic *bmiQ*-function, a prepubertal negative exponential *bmiE*-function, a nonlinear pubertal *bmiP*-function, and a stop *bmiS*-function, describing total BMI in $kg^{0.5}/m$ as a function of age:

$$bmiT(age|WHF) = bmiQ(age|WHF) + bmiE(age) + bmiP(age|WHF) - bmiS(age|WHF),$$

modified by an individual weight-height-factor (*WHF*) as shown in **Figure S1.** *WHF*=0 stands for a normal body constitution, *WHF*>0 for heavier and *WHF*<0 for leaner body constitution. The *WHF* has no influence on the *bmiE*-function.

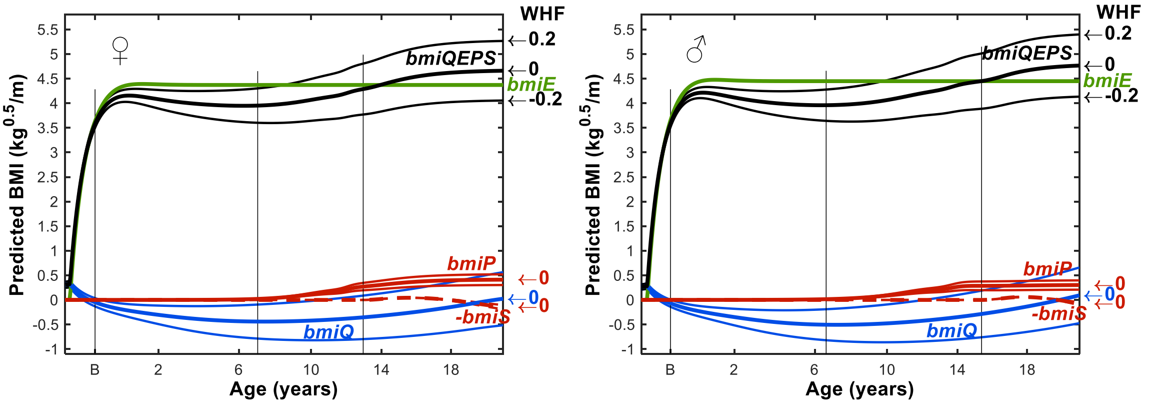

Figure S1: Total BMI (*bmiQEPS*) in kg$^{0.5}$/m for normal, heavy and lean body constitution with four growth functions *bmiQ*, *bmiE*, *bmiP* and *bmiS*.

bmiQ is derived from height and weight quadratic growth functions, *bmiE* is derived from height and weight negative exponential growth functions, *bmiP* is derived from height and weight pubertal growth functions and *bmiS* is a stop function modelling end of growth for function *bmiQ*.

The weight-height-factor (*WHF*) can modify the growth functions *bmiQ*, *bmiP* and *bmiS*, but not *bmiE*. The *bmiQEPS*-function is shown for normal (*WHF*=0, thick solid lines), heavy (*WHF*=0.2) and lean (*WHF*=−0.2) body constitution.

Birth (B), start of *bmiP,* and start of *bmiS*-function are marked with a vertical line. The chronological age scale is transformed hyperbolically to stretch the early growth period.

The QEPS BMI functions are derived from basic height and weight shape-invariant growth functions in such a way that resulting BMI functions would be additive.

The QEPS basic height functions (**Figure S2**, left panel), describing total height in cm as a function of age, are

$$hT(age) = hQ(age) + hE(age) + hP(age) - hS(age),$$

and the QEPS basic weight functions (**Figure S2**, right panel), describing total weight in kg$^{0.5}$ as a function of age, are

$$wT(age|WHF) = wQ(age|WHF) + wE(age) + wP(age|WHF) - wS(age|WHF).$$

The *WHF* is a constant of proportionality with units (kg$^{0.5}$/kg$^{0.5}$). Multiplied with the predicted individual *QPS* weight function the *WHF* is quantifying the proportion of predicted *QPS* that is added to (*WHF* positive) or subtracted from (*WHF* negative) the predicted total weight function for normal body constitution to obtain the total weight function adjusted for constitution, so QEPS weight = (QEPS predicted weight) + (QPS predicted weight) * *WHF*. Written more formally the total individual weight function describing weight in kg$^{0.5}$ is: $wT(age|WHF) = wT(age|WHF=0) + wQPS(age|WHF=0) * WHF$. For a normal body constitution *WHF*=0 and the corresponding predicted total weight function $wT(age|WHF=0)$ is equal to the individual weight function as predicted by height.

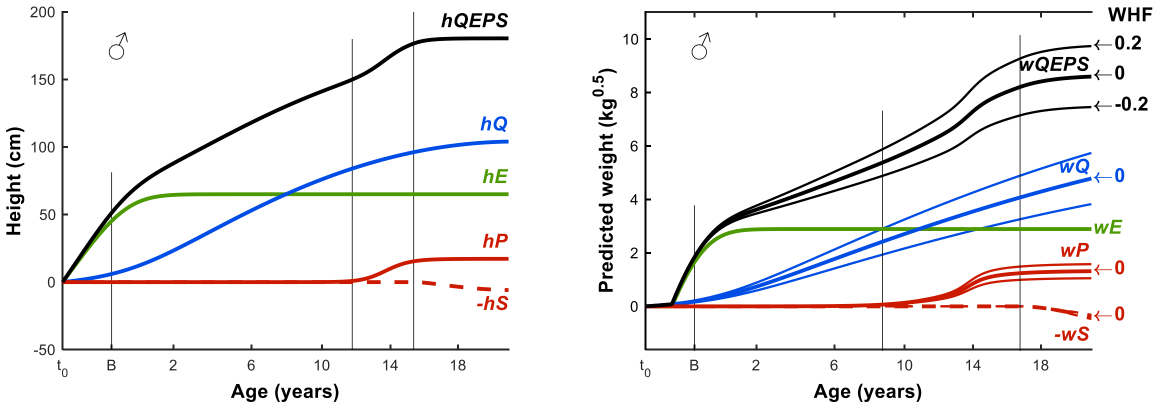

Figure S2: Total height (*hQEPS*) in cm (left panel) and total weight (*wQEPS*) in $kg^{0.5}$ for normal (WHF=0), heavy (WHF=0.2) and lean (WHF=–0.2) body constitution (right panel).

Total height in cm with *hQEPS*=*hQ*+*hE*+*hP*−*hS* (left panel) and total weight in $kg^{0.5}$ for normal, heavy, and lean body constitution with *wQEPS*=*wQ*+*wE*+*wP*−*wS* (right panel). *hQ* and *wQ* are quadratic growth functions, *hE* and *wE* negative exponential growth functions, *hP* and *wP* pubertal growth functions and *hS* and *wS* stop functions modelling end of growth for functions *hQ* and *wQ*.

The weight-height-factor (*WHF*) can modify the growth functions *wQ*, *wP* and *wS*, but not *wE*. The *wQEPS*-function is shown for the typical mean "individual" weight function, as predicted by the typical mean height function of the QEPS height model (1), with normal (*WHF*=0, thick solid lines), heavy (*WHF*=0.2) and lean (*WHF*=−0.2) body constitution. The theoretical *WHF* values 0.2 (with 20% higher *wQ*, *wP*, *wS* function) and −0.2 (with 20% lower *wQ*, *wP*, *wS* function) are chosen within the observed range of fitted *WHF* values.

Birth (B), start of *hP* and of *hS*-function, and of *wP* and of *wS*-function are marked with vertical lines. The chronological age scale is transformed hyperbolically to stretch the early growth period. The *wE* function starts at 16.6 weeks GA for girls and 17.0 weeks for boys, *hE*, *hQ* and *wQ* functions start at t_0 (about 6 weeks GA).

The QEPS BMI functions are defined in $kg^{0.5}/m$ as:

$$bmiT(age|WHF) = wT(age|WHF) \;/\; (hT(age)/100),$$

$$bmiQES(age|WHF) = wQES(age|WHF) \;/\; (hQES(age)/100),$$

$$bmiP(age|WHF) = bmiT(age|WHF) - bmiQES(age|WHF),$$

$$bmiQE(age|WHF) = wQE(age|WHF) \;/\; (hQE(age)/100),$$

$$bmiS(age|WHF) = bmiQE(age|WHF) - bmiQES(age|WHF),$$

$$bmiE(age) = wE(age) \;/\; (hE(age)/100),$$

$$bmiQ(age|WHF) = bmiQE(age|WHF) - bmiE(age|WHF),$$

$$bmiQEP_AS(age|WHF) = wQEP_AS(age|WHF) \;/\; ((hT(age) - \beta PBhsc * hP(age))/100),$$

with $\beta PBhsc$=[0.1324334;0.4074699] for [Girls; Boys], see supplement (2).

Differences in timing and amplitude between pubertal height and pubertal weight functions result in an undulation of the total BMI function *bmiT* and corresponding BMI velocity function *bmiT'*, as illustrated in detail in **Figure S3**.

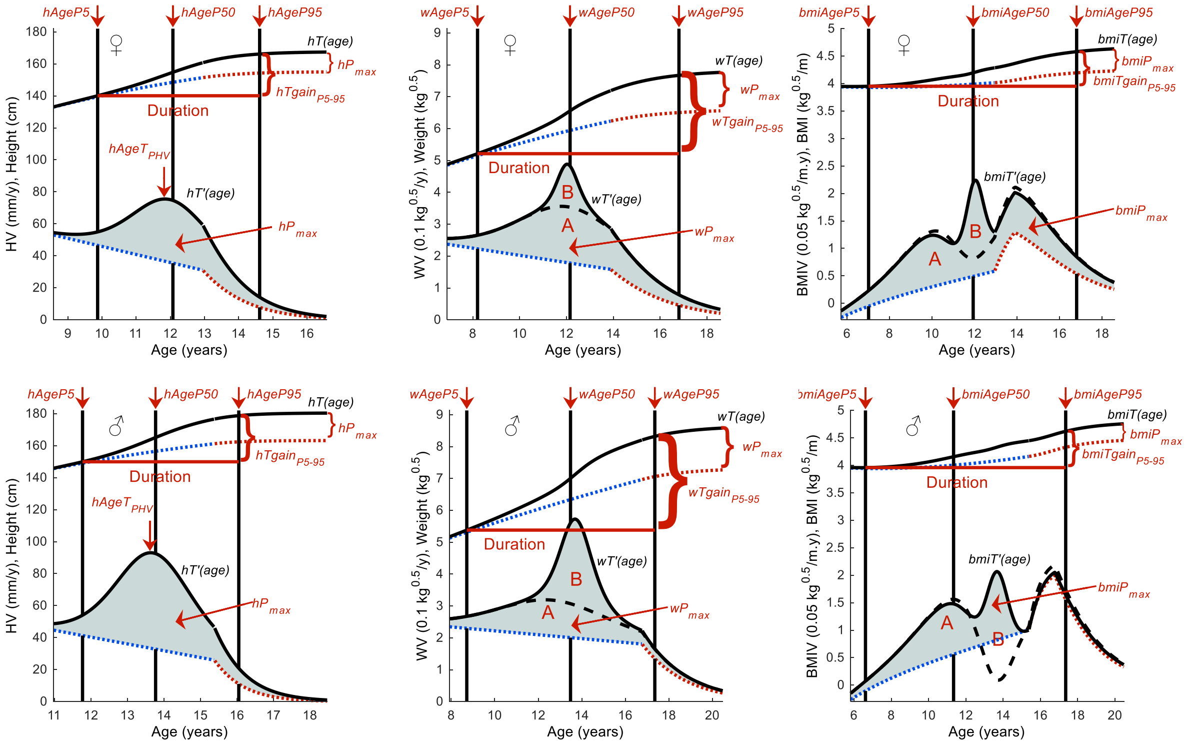

Figure S3: Detailed QEPS pubertal growth estimates for height (left panels) weight, (middle panel) and BMI (right panels) for girls (uppers panel) and boys (lower panels) during puberty

Typical mean growth functions during puberty for height and height velocity (left panels), weight and weight velocity (middle panels) and BMI and BMI velocity (right panels) for girls (upper panels) and boys (lower panels). Onset, *hAgeP5* for height, *wAgeP5* for weight and *bmiAgeP5* for BMI, as age at which 5% of the *P*-function growth is reached, mid puberty, *hAgeP50* for height, *wAgeP50* for weight and *bmiAgeP50* for BMI, as age at which 50% of the *P*-function growth is reached and Age at Peak Height Velocity $hAgeT_{PHV}$, of the total height function *hT* from the QEPS height model is reached and end of pubertal growth*, hAgeP95* for height, *wAgeP95* for weight and *bmiAgeP95* for BMI, as age at which 95% of the *P*-function growth is reached are marked with vertical lines. The duration of puberty for height, weight and BMI is indicated with horizontal lines. The pubertal height, weight or BMI gain is shown as the growth from *AgeP5* to *AgeP95* from the total growth curve ($Tgain_{P5\text{-}95}$), and from the specific pubertal part (*Pmax*); both shown at the top of the figure in the height, weight, and BMI graphs and as the marked area in the height, weight, and BMI velocity graphs.

The marked areas in the weight velocity graphs show that *wP* is a sum of two subfunctions, *wPA* and *wPB;* different for girls, with more *wPA*, than for boys, with more *wPB*. The blue dotted lines in the height and weight velocity graphs show the linear decrease of the *hQE'*and *wQE'* functions followed by red dotted lines showing the exponential decrease of the *hS'* and *wS'* functions. In the BMI velocity graph, the red dotted *bmiS'* function is first increasing by the decrease of *hS'* and then somewhat later decreasing by the decrease of the *wS'* function. Differences in timing and amplitude between pubertal height and pubertal weight functions result in an undulation of the total BMI function *bmiT* and corresponding BMI velocity function *bmiT'*.

bmiQEPS method for reference development

References for age were constructed for both total BMI and prepubertal BMI and were computed for the QEPS BMI reference population in two steps comparable to the 'QEPS method' used for the references with QEPS height functions ((3), in corresponding supplement), here replaced by corresponding QEPS BMI functions.

Special pubertal growth-aligned references *(bmiQEPSpub=bmiTpub*, *bmiQESpub* and *bmiPpub)* were constructed by aligning individual function values for *bmiT(age)*, *bmiQES(age)* and *bmiP(age)* by the individual age at onset of the pubertal growth spurt, defined as *hAgeP5*, the age at which 5% of height specific *hP*-function growth was reached (see Figure S3, left panels).

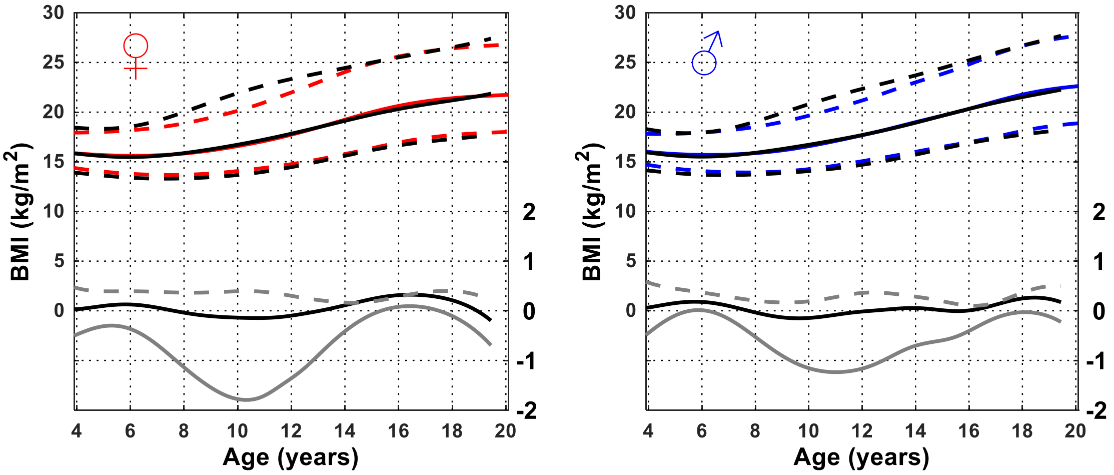

Figure S4: Total QEPS BMI vs Total LMS BMI

Comparison of the total 1990 QEPS BMI reference (red/blue) with the published (4) 1990 LMS BMI reference (black).

Total BMI references for children aged 4-18 years are shown for girls on the left and for boys on the right. The difference between the total BMIs obtained by the two methods, QEPS-derived or LMS-derived (4), are shown in the bottom of each figure. Differences in mean (solid black line), in +2SDS (solid grey line) and in −2SDS (dotted grey line) are shown.

Visual inspection shows that, although the mean curves based on the QEPS and LMS methods are quite similar, lines representing ±2SDS of the QEPS-derived reference are closer to the mean curves than lines of the LMS-derived reference. The variation of the QEPS-derived BMI reference becomes smaller since it is based on fitted height and weight functions, excluding all sorts of height and weight error, whereas the LMS-derived reference is fitted on estimated BMI values, including such errors.

REFERENCES

1. Nierop AF, Niklasson A, Holmgren A, Gelander L, Rosberg S, Albertsson-Wikland K. Modelling individual longitudinal human growth from fetal to adult life - QEPS I. J Theor Biol. 2016; 406:143-65

2. Albertsson-Wikland K, Niklasson A, Gelander L, Holmgren A, Nierop AFM. Novel type of references for weight aligned for onset of puberty – using the QEPS growth model. BMC Pediatrics. 2021; 21:507

3. Albertsson-Wikland K, Niklasson A, Holmgren A, Gelander L, Nierop AFM. A new Swedish reference for total and prepubertal height. Acta Paediatr. 2020; 109:754-63

4. Albertsson-Wikland K, Niklasson A, Gelander L, Holmgren A, Sjoberg A, Aronson AS, Nierop AFM. Swedish references for weight, weight-for-height, and body mass index: The GrowUp 1990 Gothenburg study. Acta Paediatr. 2021; 110:537-48
